# Supplementary material for: Content-rich biological network constructed by mining PubMed abstracts
Source: BMC Bioinformatics. 2004 Oct 8;5:147. doi: 10.1186/1471-2105-5-147 (PMC528731; doi:10.1186/1471-2105-5-147)
Supplement: Additional File 2 — The original results of the above study (non-essential files are deleted to keep the file size under the limit set by BMC bioinformatics). [file 1471-2105-5-147-S2.bz2 › chilibotAdditionalFile2/dip50/2ID9194558E2/html/BCL2_RAF1.html]

 


 **BCL2** and **RAF1** 
  
Found 126 abstracts in PubMed, retrieved 50.  
 

 What does Google say? 
 PDF only 
| .edu only 

---

**Interactive relationship** (e.g. stimulation, inhibition, etc)

**Inhibitory relationship**- Results Transfection of the myeloid leukemia cell lines with dsRNA corresponding to c raf  [ **RAF1** ]  and bcl 2  [ **BCL2** ]  genes decreased the expression of Raf 1  [ **RAF1** ]  and Bcl 2  [ **BCL2** ]  proteins.  Ref: 12536201 Cancer Gene Ther, 2003
- Finally, transient transfection of a dominant negative Ras allele induced Bcl 2  [ **BCL2** ]  expression and reduced Bcl 2  [ **BCL2** ]  Raf 1  [ **RAF1** ]  association demonstrating a requirement for Ras.  Ref: 12488548 Mol Pharmacol, 2003
- trans Resveratrol reverses phosphorylation of Bcl 2  [ **BCL2** ]  induced by paclitaxel and concomitantly blocks Raf 1  [ **RAF1** ]  phosphorylation, also observed after paclitaxel exposure, thus suggesting that Bcl 2  [ **BCL2** ]  inactivation may be dependent on the activation of the Raf  [ **RAF1** ]  Ras cascade.  Ref: 12510025 Neurochem Int, 2003
- C Raf  [ **RAF1** ]  cooperates with Bcl 2  [ **BCL2** ]  Bcl XL in suppression of apoptosis by a mechanism that involves targeting of C Raf  [ **RAF1** ]  to the outer mitochondrial membrane and inactivation of the pro apoptotic protein Bad.  Ref: 12079506 BMC Cell Biol, 2002
- Transfection of human embryonic 293 cells with a kinase defective Raf 1  [ **RAF1** ]  or a kinase defective mitogen activated protein kinase extracellular signal regulated kinase kinase 1 MEKK 1 suppressed Bcl 2  [ **BCL2** ]  mediated IKK beta activity and NF kappa B activation.  Ref: 12167626 J Biol Chem, 2002
- Mitochondrially active Raf 1  [ **RAF1** ]  is unable to activate extracellular signal related kinase 1 ERK1 and ERK2 but suppresses cell death by inactivating the proapoptotic Bcl 2  [ **BCL2** ]  family member BAD.  Ref: 11259582 Mol Cell Biol, 2001
- FDCP Mix BCL 2  [ **BCL2** ]  cells grown in IL 3 were distinguished from FDCP Mix and FDCP Mix BCL X L cells by a striking reduction in cellular levels of Raf 1  [ **RAF1** ]  protein.  Ref: 12721288 J Biol Chem< MedlineTA>J Biol Chem, 2001
**Neutral relationship**- Alternatively, the role of Bcl 2  [ **BCL2** ]  could be limited to the mitochondrial translocation of Raf 1  [ **RAF1** ]  and survival signaling by Raf 1  [ **RAF1** ]  is Bcl 2  [ **BCL2** ]  independent.  Ref: 11521192 Oncogene, 2001
- Replacement of the BCL 2  [ **BCL2** ]  BH4 domain with the related BCL X L BH4 sequence resulted in a switch of FDCP Mix BCL 2  [ **BCL2** ]  to erythroid fate accompanied by persistence of Raf 1  [ **RAF1** ]  protein expression.  Ref: 12721288 J Biol Chem< MedlineTA>J Biol Chem, 2001
- Thus, the antiapoptotic PI3K Akt and Bcl 2  [ **BCL2** ]  Bcl XL proteins can interact with the growth promoting Raf  [ **RAF1** ]  MEK ERK pathway and annul the cytokine dependence of certain hematopoietic cells.  Ref: 11531015 Cancer Detect Prev, 2001
- Taken together, these findings show that increased expression of p27 and Bcl 2  [ **BCL2** ]  is concomitant with altered association between Ras, Raf 1  [ **RAF1** ]  and Bcl 2  [ **BCL2** ]  and suggest that this is responsible for the growth inhibitory properties of FTI 277.  Ref: 12488548 Mol Pharmacol, 2003
- These effects are associated with differential regulation of Raf 1  [ **RAF1** ]  expression, perhaps involving the previously identified interaction between BCL 2  [ **BCL2** ]  BH4 and the catalytic domain of Raf 1  [ **RAF1** ] .  Ref: 12721288 J Biol Chem< MedlineTA>J Biol Chem, 2003
- In this report, we provide the first direct evidence that Bcl 2  [ **BCL2** ]  activates NF kappa B by a signaling mechanism that involves Raf 1  [ **RAF1** ]  MEKK 1 mediated activation of IKK beta.  Ref: 12167626 J Biol Chem, 2002
- This process requires mitochondrial translocation of Raf 1  [ **RAF1** ]  which can be achieved either by co expression of the anti apoptotic protein Bcl 2  [ **BCL2** ]  or by fusion with the transmembrane domain of the yeast outer mitochondrial membrane protein Mas 70p.  Ref: 11521192 Oncogene, 2001
- Vinblastine induced phosphorylation of Bcl 2  [ **BCL2** ]  and Bcl XL is mediated by JNK and occurs in parallel with inactivation of the Raf 1  [ **RAF1** ]  MEK ERK cascade.  Ref: 10913135 J Biol Chem, 2000
- 4 The up regulation of Fas and down regulation of Bcl 2  [ **BCL2** ]  were observed in osteoblasts expressing H Ras V12, H Ras V12 T35S, or Raf 1  [ **RAF1** ] .  Ref: 11934900 J Biol Chem, 2002
- Activation of the PI3K pathway through mp110\*ER failed to up regulate expression of the c myc, cyclin D2, cyclin D3, cyclin E, bcl 2  [ **BCL2** ] , or bcl x L genes or down regulate expression of p27 Kip1, even when coactivated with the Janus kinases Jak or the Raf  [ **RAF1** ]  Erk pathway.  Ref: 11509615 J Immunol, 2001
- Overexpression of the antiapoptotic Bcl 2  [ **BCL2** ]  protein synergized with activation of the Raf  [ **RAF1** ]  MEK ERK cascade and increased the efficiency of transformation of FDC PI and TF 1 cells.  Ref: 11531015 Cancer Detect Prev, 2001

**Non-interactive relationship** (e.g. studied together, co-existance, homology, etc.)

- To test for the mutual requirement of Raf 1  [ **RAF1** ]  and Bcl 2  [ **BCL2** ]  in apoptosis suppression the individual proteins were singly tested for survival activity in a genetic background which precludes the expression of the other.  Ref: 11521192 Oncogene, 2001
- In the absence of doxorubicin, overexpression of Bcl 2  [ **BCL2** ]  or a constitutively active form of Raf 1  [ **RAF1** ]  in MCF 7 cells did not affect proliferation rate.  Ref: 12631622 Clin Cancer Res, 2003
- Using U937 leukemic cells, we evaluated the effect of docetaxel on phosphatidylcholine PC and its metabolites, phosphatidic acid PA and diacylglycerol DAG, and their impact on MAPK and NF kappa B activation, as well as on Raf 1  [ **RAF1** ]  and Bcl 2  [ **BCL2** ]  phosphorylation.  Ref: 12724857 J Exp Ther Oncol< MedlineTA>J Exp Ther Oncol, 2003
- In addition, increased phosphorylation of Raf  [ **RAF1** ] , MEK1 2 and Bcl 2  [ **BCL2** ]  was observed in MAD2 overexpressing cells in response to vincristine.  Ref: 12527913 Oncogene, 2003
- In addition, biochemical examinations revealed that docetaxel could induce phosphorylation of both bcl 2  [ **BCL2** ]  and c raf  [ **RAF1** ]  1, but these changes were inhibited when tumor cells were pretreated or simultaneously treated with doxorubicin.  Ref: 10999771 Clin Cancer Res, 2000
- However, apoptosis suppression by C Raf  [ **RAF1** ]  also occurs in cells lacking expression of Bad or Bcl 2  [ **BCL2** ] .  Ref: 12079506 BMC Cell Biol, 2002
- These observations suggest both independent and overlapping roles for Raf 1  [ **RAF1** ]  and Bcl 2  [ **BCL2** ]  oncogenes in the resistance to growth inhibition by doxorubicin.  Ref: 12631622 Clin Cancer Res, 2003
- In recent and current clinical trials in patients with urological tumors antisense agents targeted against c raf  [ **RAF1** ]  kinase, protein kinase C alpha, protein kinase A and bcl 2  [ **BCL2** ]  are being evaluated.  Ref: 12050550 J Urol, 2002
- Here we show that apoptosis triggered by Raf  [ **RAF1** ]  depletion cannot be overcome by ectopic Bcl 2  [ **BCL2** ]  expression and occurs in the absence of cytochrome c release, arguing against a direct impact of c Raf  [ **RAF1** ]  on mitochondrial pathways of apoptosis regulation.  Ref: 12584738 Int J Cancer, 2003
- This precluded mitotic arrest, Bcl 2  [ **BCL2** ]  and Raf 1  [ **RAF1** ]  phosphorylation, and diminished cell death caused by PTX.  Ref: 12432259 Cancer Biol Ther< MedlineTA>Cancer Biol Ther, 2003
- Our data thus suggest the existence of two separate pathways of survival signaling at the mitochondria controlled either by Bcl 2  [ **BCL2** ]  or by Raf 1  [ **RAF1** ] .  Ref: 11521192 Oncogene, 2001
- Moreover, increased expression of Bcl 2  [ **BCL2** ]  protein was observed in HepG2 and Huh7 cells treated with FTI 277, and this was coincidental with reduced association between Raf 1  [ **RAF1** ]  and Bcl 2  [ **BCL2** ] .  Ref: 12488548 Mol Pharmacol, 2003
- One antisense compound has been approved for local treatment of cytomegalovirus induced retinitis, and several others are in clinical trials, including those targeting the mRNA of Bcl 2  [ **BCL2** ] , protein kinase C alpha PKC alpha, c raf  [ **RAF1** ]  or Ha ras.  Ref: 12867066 Lung Cancer, 2003
- To clarify the mechanism of Bcl 2  [ **BCL2** ]  independent survival control by C Raf  [ **RAF1** ]  at the mitochondria a search for novel mitochondrial targets was undertaken that identified voltage dependent anion channel VDAC, a mitochondrial protein porin involved in exchange of metabolites for oxidative phosphorylation.  Ref: 12079506 BMC Cell Biol, 2002
- Methods Four myeloid leukemia cell lines HL 60, U937, THP 1, and K562 were transfected with dsRNA duplexes corresponding to the endogenous c raf  [ **RAF1** ]  and bcl 2  [ **BCL2** ]  genes and the gene expression inhibition was assessed.  Ref: 12536201 Cancer Gene Ther, 2003
- Cryptophycin 52 induced phosphorylation of c  **raf1**  and bcl 2  [ **BCL2** ]  and or bcl x to comparable levels in all cell lines studied, and LNCaP cells overexpressing bcl 2  [ **BCL2** ]  were more resistant to cryptophycin 52 induced apoptosis.  Ref: 12473608 Clin Cancer Res, 2002
- Induction of Raf 1  [ **RAF1** ]  activity in MCF 7 cells overexpressing Bcl 2  [ **BCL2** ]  resulted in greater doxorubicin resistance than induction of Raf 1  [ **RAF1** ]  activity in MCF 7 cells lacking Bcl 2  [ **BCL2** ]  overexpression.  Ref: 12631622 Clin Cancer Res, 2003
- MAD2 induced sensitization to vincristine is associated with mitotic arrest and Raf  [ **RAF1** ]  Bcl 2  [ **BCL2** ]  phosphorylation in nasopharyngeal carcinoma cells.  Ref: 12527913 Oncogene, 2003
- The combination of Raf  [ **RAF1** ]  and either PI3K or Akt expression relieved cytokine dependence of some FL5.12 cells, and the efficiency of transformation could be enhanced further by Bcl 2  [ **BCL2** ]  or Bcl XL overexpression.  Ref: 11531015 Cancer Detect Prev, 2001
- Moreover, enforced expression of Raf 1  [ **RAF1** ]  redirected FDCP Mix BCL 2  [ **BCL2** ]  cells to an erythroid fate, and prohibited generation of myeloid cells.  Ref: 12721288 J Biol Chem< MedlineTA>J Biol Chem, 2001
- Conclusions RNAi is a functional pathway in human myeloid leukemia cell lines and combined RNAi of c raf  [ **RAF1** ]  and bcl 2  [ **BCL2** ]  genes may represent a novel approach to leukemia, providing a means to overcome the resistance to chemotherapeutic agents and ultimately to augment the efficacy of chemotherapy in myeloid leukemia.  Ref: 12536201 Cancer Gene Ther, 2003
- Combined RNAi for c raf  [ **RAF1** ]  and bcl 2  [ **BCL2** ]  induced apoptosis in HL 60, U937, and THP 1 cells and increased chemosensitivity to etoposide and daunorubicin.  Ref: 12536201 Cancer Gene Ther, 2003
- Reversion of the mutant phenotype could be achieved in either case by introducing a functional bcl 2  [ **BCL2** ]  gene or a mitochondria targeted version of oncogenic Raf 1  [ **RAF1** ] , demonstrating that each protein by itself is sufficient to confer protection.  Ref: 11521192 Oncogene, 2001
- The results obtained in these studies demonstrate that ablation of Raf 1  [ **RAF1** ]  or Bcl 2  [ **BCL2** ]  expression in fibroblast cells significantly increases the sensitivity towards doxorubicin induced cell death.  Ref: 11521192 Oncogene, 2001
- The ways the tumor suppressor protein p53, as well as the oncogenes ras and raf  [ **RAF1** ] , c myc and Bcl 2  [ **BCL2** ]  can influence this process at different stages are presented.  Ref: 11302264 Q J Nucl Med, 2000
- We have used a transgenic mouse model to examine the role of a candidate susceptibility gene, bcl 2  [ **BCL2** ] , for development of Raf  [ **RAF1** ]  oncogene induced lung adenomas.  Ref: 12414660 Cancer Res, 2002
- At least four of these compounds are currently in phase II trials, including those targeting protein kinase C alpha, bcl 2  [ **BCL2** ] , c raf  [ **RAF1** ] , and the R1 alpha subunit of protein kinase A.  Ref: 11122821 Curr Oncol Rep, 2000
- Although the state of 17 AAG mediated decline in Akt, c Raf  [ **RAF1** ]  1 and c Src protein levels was blunted, the totalt decline was not compromised in HL 60 Bcl 2  [ **BCL2** ]  and HL 60 Bcl xL cells.  Ref: 12623837 Blood< MedlineTA>Blood, 2000
- Thus, we propose that H Ras signals followed by Raf 1  [ **RAF1** ]  MAPK pathway but not PI3K not only reduces beta 1 mediated adhesion of osteoblasts to matrix proteins but induces apoptosis presumably via the Fas up regulation and Bcl 2  [ **BCL2** ]  down regulation.  Ref: 11934900 J Biol Chem, 2002
- Several oncogene products, most notably bcl 2  [ **BCL2** ] , c raf  [ **RAF1** ]  1, protein kinase C alpha, and H ras, have been evaluated as targets for therapeutic downregulation, and oligodeoxynucleotides designed to inhibit the expression of these products specifically have been studied extensively in phase I and II trials in cancer patients.  Ref: 11673691 Curr Opin Oncol, 2001
- RESULTS Here we show that even in the absence of Bcl 2  [ **BCL2** ]  Bcl XL, mitochondria targeted C Raf  [ **RAF1** ]  inhibits cytochrome c release and caspase activation induced by growth factor withdrawal.  Ref: 12079506 BMC Cell Biol, 2002
- Regulation of 17 AAG induced apoptosis role of Bcl 2  [ **BCL2** ] , Bcl xL, and Bax downstream of 17 AAG mediated downregulation of Akt, Raf 1  [ **RAF1** ]  and Src kinases.  Ref: 12623837 Blood< MedlineTA>Blood, 2002
- Of particular interest for the area of human cancer therapy, several cancer targets, including bcl 2  [ **BCL2** ] , BCR ABL, C raf  [ **RAF1** ]  1, Ha ras, c myc, PKC, PKA, p53 and MDM2, are reviewed as examples to illustrate the progress in this field and emphasize the importance of target selection and advanced antisense chemistry in the development of antisense therapy.  Ref: 12188878 Curr Cancer Drug Targets, 2001
- One antisense drug has been approved for local treatment of cytomegalovirus induced retinitis, and several antisense oligonucleotides are in clinical trials, including oligonucleotides that target the mRNA of  **BCL2** , protein kinase C alpha, and RAF  [ **RAF1** ]  kinase.  Ref: 11513935 Lancet, 2001
- Microtubule inhibitors, widely used in cancer chemotherapy, induce G2 M arrest and apoptosis and have in common the ability to stimulate Raf 1  [ **RAF1** ]  Bcl 2  [ **BCL2** ]  phosphorylation and activate c Jun NH2 terminal protein kinase JNK.  Ref: 11103805 Cancer Res, 2000
- In this study we investigated effects of Bcl 2  [ **BCL2** ]  and Raf 1  [ **RAF1** ]  on doxorubicin induced growth inhibition of MCF 7 breast cancer cells.  Ref: 12631622 Clin Cancer Res, 2003
- Growth Inhibition by the Farnesyltransferase Inhibitor FTI 277 Involves Bcl 2  [ **BCL2** ]  Expression and Defective Association with Raf 1  [ **RAF1** ]  in Liver Cancer Cell Lines.Farnesyltransferase inhibitors block the growth of tumor cells in vitro and in vivo with minimal toxicity toward normal cells.  Ref: 12488548 Mol Pharmacol, 2003
- Independent control of cell survival by Raf 1  [ **RAF1** ]  and Bcl 2  [ **BCL2** ]  at the mitochondria.  Ref: 11521192 Oncogene, 2001
- This could be achieved either by overexpression of the antiapoptotic protein Bcl 2  [ **BCL2** ]  or by targeting Raf 1  [ **RAF1** ]  to the mitochondria via fusion to the mitochondrial protein Mas p70.  Ref: 11259582 Mol Cell Biol, 2001
- Raf 1  [ **RAF1** ]  and bcl 2  [ **BCL2** ]  induce distinct and common pathways that contribute to breast cancer drug resistance.  Ref: 12631622 Clin Cancer Res, 2003
- Antisense oligodeoxynucleotides against bcl 2  [ **BCL2** ] , c raf  [ **RAF1** ]  1, and protein kinase C alpha continue to be the focus of ongoing trials.  Ref: 11673691 Curr Opin Oncol, 2001
